# Susceptibility patterns of *Candida* species collected from intensive care units in Portugal: a prospective study in 2020–2022

**DOI:** 10.1016/j.infpip.2024.100403

**Published:** 2024-09-24

**Authors:** Teresa Nascimento, João Inácio, Daniela Guerreiro, Priscila Diaz, Patrícia Patrício, Luís Proença, Cristina Toscano, Helena Barroso

**Affiliations:** a Unidade de Microbiologia Médica, Global Health and Tropical Medicine (GHTM), Instituto de Higiene e Medicina Tropical/Universidade Nova de Lisboa, Lisbon, Portugal; b Egas Moniz Center for Interdisciplinary Research (CiiEM), Egas Moniz School of Health & Science, 2829-511, Caparica, Almada, Portugal; c School of Applied Sciences, University of Brighton, Brighton, United Kingdom; d Hospital Prof. Doutor Fernando da Fonseca, Amadora, Portugal; e Hospital Beatriz Ângelo, Loures, Portugal; f Centro Hospitalar Lisboa Ocidental Hospital Egas Moniz, Lisboa, Portugal

**Keywords:** *Candida*, Intensive care unit, Colonisation, Surveillance, Fluconazole, Antifungal

## Abstract

**Background:**

For *Candida* infections antifungal therapy is often empirical and mainly depends on locally antifungal surveillance data, which differs between geographic regions.

**Aims:**

To monitor the epidemiology and antifungal susceptibility of *Candida* spp. from combined axillar-groin samples in intensive care unit (ICU) patients on admission (day1, D1), day 5 (D5) and day 8 (D8).

**Methods:**

From 2020 to 2022, 675 patients from three ICUs were enrolled. *Candida* isolates were identified by MALDI-TOF MS and PCR. *In vitro* antifungals susceptibility tests (AFST) were performed for fluconazole, voriconazole, amphotericin B and anidulafungin, by concentration gradient Etest® strip technique.

**Results:**

Out of 988 swabs, 355 isolates were identified as *Candida* species from 232 patients, being 89 isolates retrieved from patients that remained colonised at D5 and D8. AFST was conducted for all *Candida* isolates. The overall rate of resistance to fluconazole was 2.7%, with 3 out of 133 *C. albicans*, 2 out of 89 *C. parapsilosis* and 2 out of 24 *C. glabrata* isolates identified as resistant. Voriconazole susceptibility was observed in 99.2% of the isolates, with only one *C. albicans* isolate identified as resistant to this triazole. All isolates were susceptible to amphotericin B and 98.5% to anidulafungin. Three *Candida* spp. exhibited resistance to anidulafungin, *C. albicans*, *C. tropicalis*, and *C. parapsilosis*.

**Conclusions:**

This study highlights the importance of *C. albicans* as a frequent coloniser and showed that antifungal resistance remains uncommon among *Candida* isolates from ICUs in Portugal. The results may contribute to better management within institutions to guide therapeutic decision making.

## Introduction

The Silent Pandemic of Antimicrobial Resistance (AMR) is a phenomenon declared by the WHO as one of the ten greatest global threats to public health facing humanity today [1]. AMR research and surveillance to date has largely focused on antibacterial, antiviral and antimalarial resistance. However, it is well known that fungal infections are also a significant contributor to AMR. Recent outbreaks of systemic fungal infections, particularly in immunocompromised populations, those due to multidrug-resistant *Candida auris*, and the COVID-19-associated ‘black fungus’ disease caused by *Mucorales* species, are examples of how fungal diseases have emerged in part due to the scarcity of effective antifungals [2].

Patients in intensive care units (ICU) are at high risk of fungal infections (especially candidiasis) due to their comorbidities, the extensive use of invasive procedures and immunosuppressive therapy. Colonisation with *Candida* can be a predictor of candidaemia among ICU patients [[3], [4], [5]].

In general, *Candida* species are susceptible to antifungal drugs and the susceptibility pattern of a particular *Candida* species is often predictable [7]. However, changes in fungal epidemiology have been observed with an increase in non-*albicans* species [8,9]. This change in aetiology is also reflected in the emergence of *Candida* species with intrinsic and acquired resistance profiles to the main antifungal agents used in clinical practice. Likewise, a significant increase in the use of antifungal agents in regimens for prophylaxis and empirical therapy, has resulted in the emergence of resistant clinical isolates, particularly against triazoles (mainly fluconazole) [13,14]. Given the increase in resistance, *in vitro* antifungal susceptibility testing plays an increasingly important role in guiding therapeutic decisions [15].

By knowing the epidemiology of *Candida* colonisation and the prevalence of resistance patterns, healthcare providers can tailor empiric antifungal regimens more effectively, thus minimising the risk of treatment failure and the emergence of further antifungal drug resistance. The aim of this study was to evaluate the antifungal susceptibilities of isolates from ICU patients between 2020 and 2022 for the four most frequently used antifungals.

## Methods

### Patient characteristics

This research was conducted as a multicentre prospective observational study in the Lisbon metropolitan area between January 2020 and December 2022. Two tertiary hospitals participated in this study: *Prof. Doutor Fernando Fonseca Hospital* (FFH) and *Beatriz Ângelo Hospital* (BAH). FFH is an 802-bed tertiary hospital with an adult intensive care unit comprising a 20-bed Intensive Medicine Service unit, a 6-bed surgical unit and a 14-bed medical (multispecialty) unit and provides medical assistance to a mixed urban and rural population of around 700,000 people. BAH has an inpatient capacity of 424 beds and serves a population of approximately 300,000 residents.

Bilateral swabs were collected from the axilla/groin of patients on admission to ICU (D1) and subsequently,on day 5 (D5) and day 8 (D8). Patient data were collected usinga a form comprising epidemiological and clinical information. Exclusion criteria for the study were individuals under the age of 18 years, pregnant women and patients with mental disabilities.

## Ethics statement

The study was conducted in accordance with the Declaration of Helsinki and approved by the Institutional Ethics Committee of Hospital Prof. Doutor Fernando Fonseca (59/2019, 13/11/2019) and the Institutional Ethics Committee of Hospital Beatriz Ângelo (3655/2021, 21/07/2021). Informed consent was obtained from all subjects involved in the study.

### Identification of isolates

Isolates were identified to the species level using culture-based methods. All isolates were analysed with MALDI-TOF MS—VITEK® MS (bioMérieux, Marcy l’Etoile, France) using VITEK® MS v3.2 software. In addition, isolates were tested with a *C. auris*-specific PCR assay and screened for *Candida* cryptic species from the main complexes [16,17].

### Antifungal susceptibility testing

Minimum inhibitory concentration (MIC) assays were performed for amphotericin B, anidulafungin, fluconazole and voriconazole, with concentrations between 0.002–32 mg/L using the E-test® method (bioMérieux, Marcy l'Etoile, France/HiMedia, Maharashtra, India). The testing procedure was carried out with slight changes from the method recommended by the manufacturer. We used RPMI 1640® agar plates (HiMedia, Maharashtra, India) and gradient diffusion strips were incubated at 35°C for 24 and 48 hours. *C. parapsilosis* (ATCC 22019) and *C. krusei* (ATCC 6258) reference strains were used as quality controls. MIC50 and MIC90 were defined as the minimum inhibitory concentration (MIC) required to inhibit the growth of 50% and 90% of the organisms, respectively. MIC results were interpreted based on the clinical breakpoints recommended by the European Committee on Antimicrobial Susceptibility Testing (EUCAST) and isolates were assigned to the clinical categories “susceptible” (S), “susceptible, increased exposure” (I) and “resistant”(R) [18,19]. In the absence of defined clinical breakpoints for some *Candida* species and/or antifungal molecules, epidemiological cut-off values (ECOFFs) were used to discriminate wild type (WT) (WT; MIC ≤ ECOFF) and non-WT (NWT) (MIC > ECOFF) [18,20].

For fluconazole, *C. albicans*, *C. parapsilosis* and *C. tropicalis* isolates with MIC ≤2 mg/L and >4 mg/L were categorized as sensitive (S) and resistant (R), respectively; *Nakaseomyces glabrata* (*Candida glabrata)* isolates with MIC ≥16 mg/L were considered as R; *Meyerozyma guilliermondii* (*Candida guilliermondii*) isolates were considered WT when MIC ≤16 mg/L (ECOFF) [21]. Voriconazole MIC interpretation for *C. albicans* was MIC ≤0.06 mg/L (S) and ≥0.25 mg/L (R); for *C. parapsilosis* and *C. tropicalis* isolates was MIC ≤0.125 mg/L (S) and ≥0.250 mg/L (R); *C. glabrata* isolates were considered WT when MIC ≤1.0 mg/L (ECOFF). For anidulafungin, MIC interpretation was MIC ≥ 0.03 mg/L (R) for *C. albicans*; MIC ≥ 0.06 mg/L (R) for *C. tropicalis* and *C. glabrata* and MIC ≥ 4 mg/L (R) for *C. parapsilosis*. For amphotericin B, MIC interpretation was MIC ≤1.00 mg/L (S) for *C. albicans*, *C. parapsilosis*, *C. glabrata* and *C. tropicalis* isolates. *Clavispora lusitaniae* (*Candida lusitaniae*) and *C. guilliermondii* isolates were considered WT when MIC ≤0.5 mg/L (ECOFF) [22].

### Statistical analysis

The demographic, clinical and mycological characteristics of the study group were organised in a database and data were analysed using IBM SPSS Statistics v. 29.0 (IBM Corp., Armonk, NY) software. Data analysis was carried out by using descriptive and inferential methodologies. Comparison of the demographic and clinical characteristics of colonised patients at admission, as a function of the ICU, was performed using the Chi-square test. A *P*-value < 0.05 was taken to be statistically significant for all the above inferential analyses.

## Results

### Demographic and clinical characteristics of patients

In the study period, 2020–22, 675 patients were enrolled, comprising 135 patients in 2020 (71 and 64 patients from medical and chirurgical FFH ICU, respectively), 170 patients in 2021 and 370 in 2022 (540 patients from BAH ICU). A decrease in yeast colonisation was observed since the beginning of the study, with 47.4% (64 out of 135) of the patients colonised in 2020, 38.8% (66 out of 170) in 2021 and 27.6% (102 out of 370) in 2022.

Colonised patients were mainly male, but the difference was not significant (*P*= 0.124). The mean age was 63 years old, ranging from 18 to 93 years old. Underlying comorbidities, such as pulmonary infection (*P*=<0.001), haematological malignancies (*P*=0.002) and anaemia (*P*= 0.014) were significantly associated with patients, respectively in medical FFH, surgical FFH and general BAH ICU unit. Additionally, the use of antibiotics (*P* < 0.001) and antiviral therapy *P* < 0.001) was significantly higher among colonised patients in the medical FFH ICU. The complete demographic and clinical characteristics of the colonised patients is reported in Table I.

**Table I:** Demographic and clinical characteristics of colonised patients at admission by hospital ICU^a^

Patient characteristics	All patients (n=232)	Medical FFH ICU (n=38)	Surgical FFH ICU (n=26)	BAH ICU (n=168)	*P*
**Age (years)**					0.215
18–40	25 (10.8)	6 (15.8)	2 (7.7)	17 (10.1)	
41–60	56 (24.1)	13 (34.2)	4 (15.4)	39 (23.2)	
61–80	120 (51.7)	15 (39.5)	19 (73.1)	86 (51.2)	
81+	31 (13.4)	4 (10.5)	1 (3.8)	26 (15.5)	
**Gender**					
Male	132 (56.9)	16 (42.1)	14 (53.8)	102 (60.7)	0.106
Female	100 (43.1)	22 (57.9)	12 (46.2)	66 (39.3)	
**Underlying comorbidities**					
Pulmonary infection	70 (30.2)	24 (63.2)	7 (26.9)	39 (23.2)	**< 0.001**
Cardiovascular disease	38 (16.4)	2 (5.3)	1 (3.8)	35 (20.8)	0.012
Gastrointestinal pathology	36 (15.5)	3 (7.9)	7 (26.9)	26 (15.5)	0.119
Urinary tract infection	20 (8.6)	1 (2.6)	0 (0.0)	19 (11.3)	0.057
Solid tumour	43 (18.5)	4 (10.5)	6 (23.1)	33 (19.6)	0.349
Haematological neoplasms	5 (2.2)	0 (0.0)	3 (11.5)	2 (1.2)	**0.002**
Diabetes *mellitus*	64 (27.6)	10 (26.3)	7 (26.9)	47 (28.0)	0.976
HIV/AIDS	1 (0.4)	0 (0.0)	0 (0.0)	1 (0.6)	0.826
Anaemia (Hb <10mg/dl)	26 (11.2)	0 (0.0)	1 (3.8)	25 (14.9)	**0.014**
Severe immunodeficiency	4 (1.7)	0 (0.0)	0 (0.0)	4 (2.4)	0.461
COVID-19	7 (3.0)	0 (0.0)	0 (0.0)	7 (4.2)	0.253
**Risk factors**					
Presence of CVC^b^	138 (59.5)	23 (60.5)	21 (80.8)	94 (56.0)	0.365
Mechanical ventilation	84 (36.2)	13 (34.2)	13 (50.0)	58 (34.5)	0.299
TPN^c^	3 (1.3)	1 (2.6)	0 (0.0)	2 (1.2)	0.642
Surgery	59 (25.4)	6 (15.8)	10 (38.5)	43 (25.6)	0.123
Neutropenia	2 (0.9)	0 (0.0)	0 (0.0)	2 (1.2)	0.681
Urinary catheter	154 (66.4)	28 (73.7)	20 (78.9)	106 (63.1)	0.221
Dialysis	23 (9.9)	3 (7.9)	4 (15.4)	16 (9.5)	0.585
**Antibiotic therapy**^d^	133 (57.3)	31 (81.6)	20 (76.9)	82 (48.8)	**< 0.001**
**Fluconazole**	6 (2.6)	1 (2.6)	0 (0.0)	5 (3.0)	0.673
**Echinocandins**	2 (0.9)	1 (2.6)	1 (3.8)	0 (0.0)	0.062
**Antiviral therapy**	4 (1.7%)	4 (10.5)	0 (0.0)	0 (0.0)	**< 0.001**

a Data are presented as No. (%) unless otherwise specified.

b Central Venous Catheter (CVC).

c Total Parenteral Nutrition (TPN).

d 48 hours prior.

### Species identification and distribution

During the study period, 371 yeasts (355 *Candida* species and 16 non-*Candida* yeasts) were grown from 232 patients. *C. albic*ans was the predominant species isolated with 185/355 (52.1%), followed by *C. parapsilosis* complex 112/355 (31.5%) [*C. parapsilosis* sensu *stricto* (n=109), *C. orthopsilosis* (n=2), *C. metapsilosis* (n=1)], *C. glabrata*, 36/355 (10.1%), *C. tropicalis*, 15/355 (4.5%), *C. lusitaniae*, 4/355 (1.1%) and *C. guilliermondii*, 3/355 (0.8%) (Table II). A recurrence of colonisation with the same yeast during all length of stay (D1, D5 and D8) was observed in 89 patients, 52 colonised with *C. albicans*, 23 with *C. parapsilosis*, 12 with *C. glabrata* and two with *C. tropicalis*. The dynamics of *Candida* species isolates from 2020 to 2022 is expressed in Table II.

**Table II:** Distribution of *Candida* spp. isolated from screening swabs over time (2020–2022).^a^

Species	2020 (n= 101)	2021 (n= 100)	2022 (n= 154)	Total (n= 355)
*C. albicans*	40 (39.6)	57 (57.0)	88 (57.1)	185 (52.1)
*C. parapsilosis* complex^b^	43 (42.6)	25 (25.0)	44 (28.6)	112 (31.5)
*C. glabrata*	8 (7.9)	12 (12.0)	16 (10.4)	36 (10.1)
*C. tropicalis*	5 (5.0)	6 (6.0)	4 (4.6)	15 (4.5)
*C. lusitaniae*	3 (3.0)	0 (0.0)	1 (0.6)	4 (1.1)
*C. guilliermondii*	2 (2.0)	0 (0.0)	1 (0.6)	3 (0.8)

a Data are presented as No. (%) unless otherwise specified.

b Two isolates proved to be *C. orthopsilosis* (1.8%); one isolate proved to be *C. metapsilosis* (0.9%).

### Antifungal susceptibility profiles

Antifungal sucetibility tests were conducted on 355 *Candida* isolates. Duplicate susceptibility profiles from *Candida* species that colonised patients during their ICU stay (from Day 1 to Day 8) were removed. There remained 266 isolates to determine antifungal resistance rates using the interpretative rules defined by EUCAST [22] (Table II). MIC readings for quality control strains (ATCC 90028 and ATCC 22019) were within the limits described in EUCAST. Details of MIC range, MIC50, MIC90 are presented in Table III.

**Table III:** Minimal inhibitory concentrations (MICs) of *Candida* species from screening samples as determined according to the European Committee on Antimicrobial Susceptibility Testing (EUCAST)

*Candida* species	No. of isolates	Antifungal agents	Range	MIC50	MIC90	S	I	R	WT	NWT
*C. albicans*	133	Fluconazole	≤0.001–32	0.250	2.0	130 (97.7)	NA	3 (2.3)	115 (79.3)	27 (20.3)
		Voriconazole	≤0.001–32	0.004	0.064	126 (94.7)	6 (4.5)	1 (0.8)	115 (86.5)	18 (13.5)
		Amphotericin B	≤0.001–1.0	0.125	0.5	133 (100)	NA	0 (0.0)	133 (100)	0 (0.0)
		Anidulafungin	≤0.001–2.0	0.002	0.016	131 (98.5)	NA	2 (1.5)	131 (98.5)	2 (1.5)
*C. parapsilosis* complex	89	Fluconazole	0.016–32	0.125	1.0	87 (97.8)	NA	2 (2.2)	87 (97.8)	2 (2.2)
		Voriconazole	≤0.001–0.125	0.008	0.064	89 (100)	NA	0 (0.0)	87 (97.8)	2 (2.2)
		Amphotericin B	≤0.001–0.5	0.125	0.5	89 (100)	NA	0 (0.0)	89 (100)	0 (0.0)
		Anidulafungin	0.002–32.0	1.0	2.0	88 (98.9)	NA	1 (1.1)	88 (98.9)	1 (1.1)
*C. glabrata*	24	Fluconazole	0.125–32	2.0	32.0	NA	22 (91.6)	2 (8.3)	22 (91.6)	2 (8.3)
		Voriconazole	0.002–0.250	0.032	0.250	NA	NA	NA	24 (100)	0 (0.0)
		Amphotericin B	0.032–0.5	0.5	0.5	24 (100)	NA	0 (0.0)	24 (100)	0 (0.0)
		Anidulafungin	≤0.001–0.064	0.006	0.064	24 (100)	NA	0 (0.0)	24 (100)	0 (0.0)
*C. tropicalis*	13	Fluconazole	0.008–1.0	0.064	0.125	13 (100)	NA	0 (0.0)	13 (100)	0 (0.0)
		Voriconazole	≤0.001–0.125	0.004	0.125	13 (100)	NA	0 (0.0)	13 (100)	0 (0.0)
		Amphotericin B	≤0.001–0.5	0.064	0.50	13 (100)	NA	0 (0.0)	13 (100)	0 (0.0)
		Anidulafungin	0.004–0.250	0.008	0.125	12 (92.3)	NA	1 (7.7)	12 (92.3)	1 (6.7)
*C. lusitaniae*	4	Fluconazole	0.064–1.0	NA	NA	NA	NA	NA	NA	NA
		Voriconazole	0.008–0.125	NA	NA	NA	NA	NA	NA	NA
		Amphotericin B	0.250–4.0	NA	NA	NA	NA	NA	1 (25.0)	3 (75.0)
		Anidulafungin	0.002–0.064	NA	NA	NA	NA	NA	4 (100)	0 (0.0)
*C. guilliermondii*	3	Fluconazole	4.0–8.0	NA	NA	NA	NA	NA	3 (100)	0 (0.0)
		Voriconazole	0.001–0.016	NA	NA	NA	NA	NA	NA	NA
		Amphotericin B	0.004–0.125	NA	NA	NA	NA	NA	3 (100)	0 (0.0)
		Anidulafungin	0.002–0.004	NA	NA	NA	NA	NA	3 (100)	0 (0.0)
Total	259^a^	Fluconazole	≤0.001–32	0.250	2,0	225 (97.3)	27 (9.5)	7 (2.7)	231 (88.2)^b^	31 (11.8)^b^
		Voriconazole	≤0.001–32	0.008	0.064	252 (97.5)	6 (2.3)	1 (0.4)	237 (91.5)	22 (8.5)
		Amphotericin B	≤0.001–4.0	0.125	0.5	266 (100)	NA	0 (0.0)	266 (100)^c^	0 (0.0)^c^
		Anidulafungin	≤0.001–32.0	0.008	1	255 (98.5)	NA	4 (1.5)	262 (98.5)^c^	4 (1.5)^c^

NA: Not Available.

a Susceptibility rates did not include the four and three isolates of *C. lusitaniae* and *C. guilliermondii*, respectively.

b Total of 262 isolates.

c Total of 266 isolates.

Most *Candida* isolates were considered susceptible to triazoles. For fluconazole, resistance was observed in generally susceptible species such as *C. albicans* (2.3%) and *C. parapsilosis* (2.2%). For voriconazole, resistance was only observed for *C. albicans* (0.8%). All isolates showing a NWT phenotype to voriconazole were also fluconazole NWT. It is worth noting that the cryptic species from the *C. parapsilosis* complex were found to be susceptible to all antifungals tested. Resistance to both fluconazole and voriconazole was detected for one isolate of *C. albicans* (Table IV). All *Candida* spp. isolates were susceptible to amphotericin B. Only one of the four *C. lusitaniae* isolates was WT. Resistance to anidulafungin was observed for three species: *C. tropicalis* (7.7%), *C. albicans* (1.5%) and *C. parapsilosis* (1.1%). Overall, only 1.5% of isolates evidenced resistance to anidulafungin. The only resistant isolate of *C. tropicalis* showed resistance to echinocandins.

**Table IV:** Main demographic, clinical and microbiological characteristics of the patients with azole and echinocandins resistant *Candida* spp. (n=9)

	Patient 1 D8	Patient 2 D1/D5	Patient 3 D8	Patient 4 D1	Patient 5 D1
Hospital/Unit	FFH (Medical)	FFH (Surgical)	BAH	BAH	BAH
Gender	Male	Male	Female	Female	Female
Age (years)	77	79	62	65	79
*Candida* spp.	*C. albicans*	*C. parapsilosis*	*C. albicans*	*C. albicans*	*C. parapsilosis*
MIC [mg/L]					
Fluconazole	8.0	8.0	8.0	32	32
Voriconazole	0.250	0.125	0.125	32	0.125
Underlying diseases	Respiratory/DM	Respiratory	Oncologic	Oncologic	Cardiovascular
ICU risk factors	CVC^a^/IAV^b^/VC^c^	CVC^a^/IAV^b^/VC^c^	None	CVC^a^/IAV^b^/VC^c^	CVC^a^/IAV^b^/VC^c^/S^d^
Previous therapy					
Antibiotic	CTX^e^/Mer^f^/PIP-TZM^g^	PIP-TZM^g^	Quinolone	None	None
Antiviral	Yes	No	No	No	No
Antifungal	No	No	No	No	No
Clinical Interpretation	Colonisation	Colonisation	Colonisation	Colonisation	Colonisation
3 month outcome	NA^h^	NA^h^	Deceased	Alive	Deceased
	Patient 6 D1/D5	Patient 7 D1	Patient 8 D1		Patient 9 D1
Hospital/Unit	BAH	BAH	FFH (Medical)		BAH
Gender	Male	Male	Female		Female
Age (years)	68	41	68		79
*Candida* spp.	*C. albicans*	*C. albicans*	*C. parapsilosis*		*C. tropicalis*
MIC [mg/L]					
Anidulafungin	1.0	2.0	32		0.250
Underlying diseases	Cardiovascular	Hb <10 mg/dl	Respiratory		Respiratory
ICU risk factors	None	CVC^a^	VC^b^		VC^b^
Previous therapy					
Antibiotic	Meropenem	No	Clarithromycin/AMC^c^		No
Antiviral	Yes	No	No		No
Antifungal	No	No	No		Fluconazole
Clinical Interpretation	Colonisation	Colonisation	Colonisation		Colonisation
3 month outcome	Deceased	Deceased	NA		Alive

a Central Vascular Catheter.

b Invasive Assisted Ventilation.

c Urinary Catheter.

d Surgery.

e Cefotaxime.

f Meropenem.

g Piperacilin-Tazobactam.

h Not Available.

### Antifungal resistance phenotypes by patient

Similar antifungal susceptibility patterns were found for *Candida* isolates grown from the same patients who remained colonised throughout their ICU stay (n=89). Additionally, for the BAH ICU, in which the study was carried out continuously for 14 months, the MIC50 and MIC90 were stable overtime. The isolates from the two ICUs under study at the FFH and at BAH showed similar susceptibility profiles, respectively 94.7% and 96.0%. However, for both hospitals, resistance phenotypes were observed in *Candida* species traditionally susceptible to azoles: *C. albicans* and *C. parapsilosis* (Table IV). Among the observed resistance phenotypes, 44.4% (four out of nine) were identified in samples collected during ICU stay, although this was not found statistically significant (*P*=0.635).

The clinical and sociodemographic characteristics of the patients from which azole and anidulafungin resistant isolates were obtained are shown in Table IV. Only the presence of assisted ventilation was found to be associated with azole resistance (*P*=0.016). No statistically significant associations were found for the other predisposing factors such as underlying diseases and concomitant antimicrobial therapy.

## Discussion

We describe the results of the first prospective multicentre study in Portugal aiming to characterise, for consecutive years, the antifungal profile of *Candida* colonisation isolates from ICU on admission and for the whole length of stay.

The findings are partly in line with previous studies (carriage/infection) worldwide showing that, although *C. albicans* still ranks first as the main colonising yeast in ICU patients, a change towards NAC colonisation has been observed in the ICU in the last decade [23,24]. *Candida albicans* was the main species found in our study (52.1% of the isolates), but in a proportion not much above the non-*C. albicans* species. However, when analysed over the time, an higher prevalence of non-*albicans Candida* was found in 2020, with a decrease in 2021 and 2022 in which years the distribution of *Candida* species was similar. Nevertheless, it should be noted that the 2020 results referred to a different ICU. This could be a bias in the results, but it might also reflect the effects of infection control measures implemented during the COVID-19 pandemic, as *C. parapsilosis* ranked first in 2020. It would be interesting to see if this trend continues in the coming years.

In this study, susceptibility analysis relied on the E-test®, which has demonstrated high categorical and essential agreement with the EUCAST broth microdilution method [20].

The overall rates of resistance to fluconazole and voriconazole in our study were in line with previous reports, particularly from other European countries [25]. Nationwide fungaemia studies have reported resistance rates for fluconazole ranging from 2.7% (Iceland) to 9.4% (Denmark) [26,27]. In this study 2.3% of the *C. albicans* isolates were resistant to fluconazole, in agreement with values in the literature that refer to relatively low worldwide acquired azole drug resistance rates in this species (1–6% worldwide) [21,28]. Relatively low antifungal resistance rates were also found among our NAC isolates. Proportion of NAC isolates resistant to fluconazole ranged from 0% (*C. tropicalis*) to 8.3% (*C. glabrata*). Former European studies [[29], [30], [31], [32]] have shown an increase in azole resistance among invasive NAC species (e.g., percentages of *C. glabrata* fluconazole resistance ranged from 2.8% in Iceland [26] to 88.6% in Denmark [27]). From our epidemiological data, few patients being treated with fluconazole, in contrast with centres where azole use waswidespread and continued for long-term for prophylaxis and the treatment of infection. This mmay explain why most *C. glabrata* isolates in this study were susceptible with increased exposure. Fluconazole is considered the first option for the empirical treatment of invasive candidiasis due to *C. parapsilosis* [33]. Two *C. parapsilosis* isolates (2.2%) were found to be resistant to fluconazole in our study. These results are in line with the global fluconazole resistance rates for this species, ranging between 2 and 5% [34]. However, the emergence of fluconazole-resistant *C. parapsilosis* have been a growing concern worldwide in the past years, with studies reporting its occurrence in Colombia [12] and France [35]. The resistance is often associated with specific mutations, such as Y132F in the *ERG11* gene [12]. Resistance to azoles was not found in our study for *C. tropicalis*, but resistance rates described in other studies were as high as 4.9%, with remarkable differences between continents [21].

There were insufficient data to demonstrate correlations between MIC values for fluconazole and voriconazole against *C. guilliermondii* and *C. lusitaniae* and clinical outcomes [36]. Other authors using Clinical Laboratory Standards Institute (CLSI) criteria refer to *C. guilliermondii* as a species showing intrinsically elevated fluconazole MIC values [15], but this was not the case in our study. *C. guilliermondii* isolates were considered WT with a range of MIC 4.0–8.0 mg/L.

Susceptibility testing for amphotericin B is not routinely performed for *Candida* spp., as resistance is very uncommon apart with *C. lusitaniae* [37]. No isolates resistant to amphotericin B were identified in this study. However, it is noteworthy that the MIC90 corresponded to a high MIC value (0.5 mg/L).

The overall rate of anidulafungin resistance was 1.5% in this study, in contrast with previous reports from other Portuguese centres [38], which found higher resistance rates. Results for 20 years of the SENTRY Antifungal Surveillance Program demonstrated that echinocandin resistance did not show significant changes for the five most common *Candida* spp. [21,39]. Nationwide fungaemia studies have reported resistance rates ranging from 0% (Iceland) to 15% (Portugal) for echinocandins (caspofungin) [24,26,27,38]. In this study, the *C. tropicalis* resistance to anidulafungin of 7.7% comprised only one isolate out of 15 and it is not possible to draw clear conclusions about the resistance pattern. *C. tropicalis* may acquire resistance after short-term treatment with caspofungin, which was not observed in the patient in this study. *C. parapsilosis* tends to be more tolerant to echinocandins as a result of the natural polymorphisms present in this species [40]. The results in this study are consistent with previous studies, showing an MIC range of 0.002–32 mg/L for the *C. parapsilosis* complex, with an MIC90 of 1 mg/L [41,42].

Surveillance studies have raised attention regarding the occurrence of multi-drug resistant (MDR) profiles among both *C. albicans* and NAC isolates [43,44], which was not found in this study. No *Candida* isolates showed resistance towards more than one antifungal agent of a different class. This contrasts with previous European studies, including Portugal, that described the emergence of MDR strains in *C. glabrata*, *C. krusei*, *C. parapsilosis*, and *C. tropicalis* [25,38].

Initiating empirical antifungal therapy remains a controversial issue in the management of ICU patients. Based on these results, fluconazole is an acceptable alternative to echinocandin as initial therapy in patients who were not critically ill and had no prior azole exposure without waiting for the antifungal susceptibility *in vitro* tests.

Antifungal exposure may favour the selection of acquired resistance of *C. albicans* and NAC species [45]. As in the colonised cohort in this study, the patient data showed that antifungals were not routinely used for prophylaxis and or empirical treatment (3.5%), which could explain the lower rates of resistance, especially for fluconazole. However, an association between patients colonised by *Candida* spp. and antibiotic and antiviral therapy was observed but without expression of resistance phenotypes.

It was observed in our study that the presence of assisted ventilation was associated with azole resistance profiles. However, given the low number of patients colonised by resistant isolates, it was not possible to draw conclusions about antifungal resistance and the predisposing patient's factors. Our study focussed only on *Candida* isolates colonising patients, which might be considered a limitation as it is difficult to establish an association with infection, and thus to conclude about the clinical impact of susceptibility versus resistance profiles within these isolates.

We analysed the susceptibility pattern of isolates of *Candida* species in Portugal.The findings may be relevant worldwide. It is known that resistant species are rarely limited to specific locations. Any area with highly drug-resistant strains can act as a reservoir, from which resistant species can be transmitted to other parts of the globe through humans, water, agricultural products and animals. The information provided here can help develop treatment recommendations in the future that may need to be adapted globally. Therefore, the results obtained from this surveillance study may have not only national relevance but may also have an international impact by determining the importance of the associated health environment in promoting the transmission of resistant fungi.

## Conclusions

This study is the first characterisation of *Candida* spp. antifungal drug susceptibility, along with clinical characteristics/risk factors for *Candida* ICU colonisation in Portugal. The findings within this cohort showed that resistance remains uncommon among *Candida* isolates in the ICUs under investigation. Additionally, we support the need to have antifungal treatment guidelines based on epidemiology to choose the therapeutic options with the best antifungal coverage and least selective pressure. Ideally this should occur in the context of an antifungal stewardship program. In conclusion, these antifungal susceptibility profiles provide information on which antifungal agents are most likely to be effective against specific fungal pathogens, enabling tailored treatment strategies and informing broader infection prevention and control measures.

## Conflict of interest statement

The authors declare no conflict of interest.

## Funding statement

The authors thank FCT/MCTES for the financial support to CiiEM (10.54499/UIDB/04585/2020) through national funds.

## Credit author statement

- Conceptualization: Teresa Nascimento, João Inácio, Priscila Diaz, Patrícia Patrício, Cristina Toscano, Luís Proença, and Helena Barroso.
- Project Administration and Funding Acquisition: Helena Barroso.
- Investigation: Teresa Nascimento, Daniela Guerreiro, Cristina Toscano, João Inácio, Patrícia Patrício and Helena Barroso.
- Formal Analysis: Luís Proença.
- Supervision: João Inácio and Helena Barroso.
- Writing – Original Draft: Teresa Nascimento.
- Writing – Review & Editing: Teresa Nascimento, João Inácio, Luís Proença, Cristina Toscano, and Helena Barroso.
